# Lipidomics Analysis Explores the Mechanism of Renal Injury in Rat Induced by 3-MCPD

**DOI:** 10.3390/toxics11060479

**Published:** 2023-05-25

**Authors:** Tao Wei, Na Cao, Tiantian Han, Yi Chen, Xingtao Zhou, Liyang Niu, Wenting Liu, Chang Li

**Affiliations:** 1 State Key Laboratory of Food Science and Technology, Nanchang University, Nanchang 330047, China; wtnever@163.com (T.W.); cn170309@163.com (N.C.); ht1830266128@163.com (T.H.); chenyi-417@163.com (Y.C.); zhouxingtao@ncu.edu.cn (X.Z.); niyang@outlook.com (L.N.); liuwenting1207@163.com (W.L.); 2 China-Canada Joint Laboratory of Food Science and Technology (Nanchang), Nanchang University, Nanchang 330047, China; 3 Key Laboratory of Bioactive Polysaccharides of Jiangxi Province, Nanchang University, Nanchang 330047, China

**Keywords:** 3-MCPD, nephrotoxicity, lipidomics, biomarkers, pathway

## Abstract

3-monochloropropane-1,2-diol (3-MCPD) is a food-process toxic substance, and its main target organ is the kidney. The present study examined and characterized the nephrotoxicity and the lipidomic mechanisms in a model of kidney injury in Sprague Dawley (SD) rats treated with high (45 mg/kg) and low (30 mg/kg) doses of 3-MCPD. The results showed that the ingestion of 3-MCPD led to a dose-dependent increase in serum creatinine and urea nitrogen levels and histological renal impairment. The oxidative stress indicators (MDA, GSH, T-AOC) in the rat kidney altered in a dose-dependent manner in 3-MCPD groups. The lipidomics analysis revealed that 3-MCPD caused kidney injury by interfering with glycerophospholipid metabolism and sphingolipid metabolism. In addition, 38 lipids were screened as potential biomarkers. This study not only revealed the mechanism of 3-MCPD renal toxicity from the perspective of lipidomics but also provided a new approach to the study of 3-MCPD nephrotoxicity.

## 1. Introduction

Since 1978, researchers detected chloropropanols in acid-hydrolyzed vegetable protein (HVP) solution [1]. Research on chloropropanol was a hot topic, especially 3-chloro-1,2-propanediol (3-MCPD), which received more attention because of its higher content than other isomers. In 2004, it was found that the content of 3-MCPD esters in food were much higher than that of free 3-MCPD. 3-MCPD esters are widely present in edible oils [2]. In addition to refined edible oils, they are also present in other foods, such as bread, coffee, infant formula, malt, milk powder and fried potato chips [3,4,5,6], and they were even detected in human breast milk [7]. 3-MCPD esters could be efficiently enzymatically digested into free 3-MCPD in the intestine [8]. In 2017, WHO reported that adults and children daily exposure of 3-MCPD ranged from 0.2 to 1.7 μg/kg bw per day, while the maximum daily exposure of infants was 10 μg/kg bw per day [9].

3-MCPD is considered mutagenic and carcinogenic (Group 2B) [10]. 3-MCPD has toxic effects on the kidney, testis, liver, brain, thymus and lung [11,12,13]. Numerous toxicological studies showed that the kidneys are most susceptible to the toxic effects of 3-MCPD [14,15,16]. The researchers found that intragastric administration of a daily dose of 30 mg/kg of 3-MCPD for 28 days significantly increased the serum creatinine and blood urea nitrogen levels in rats, and the pathological examination revealed that some collecting duct epithelial cells were degenerated and swollen, and renal tubular epithelial cells were shed [17]. The pathological examination also suggested glomerular atrophy and tubular lumen enlargement after 28 days of 40 mg/kg 3-MCPD gavage [18]. In addition, after administration of 100 mg/kg 3-MCPD to mice, it was found that the weight of the mice continued to decrease, and the kidney index increased significantly after 7 days. Severe oxidative stress was observed in the kidney after X-Gal staining of the kidney segment [11].

Regarding the mechanism of nephrotoxicity caused by 3-MCPD, Peng et al. found that free 3-MCPD induced apoptosis in human kidney cells by downregulating Bcl2 expression, releasing cytochrome C from mitochondria into the cytoplasm, and activating the downstream apoptosis promoters Caspase-9 and Caspase-3, Caspase-6 and Caspase-7 [19]. Based on proteomics, Yang et al. explored the kidney toxic effects of 3-MCPD 1-monooleate and 1-monostearate on the kidney of SD rats, and pointed that the 3-MCPD esters influenced proteins involved in the pathways for apoptosis, ion transportation, the metabolism of xenobiotics, as well as enzymes related to endogenous biological metabolisms of carbohydrates, nitrogen, amino acids, lipids, fatty acids, and the tricarboxylic acid (TCA) cycle, providing partial explanation for the nephrotoxicity of 3-MCPD esters [20]. 3-Chloropropanol-1-palmitate monoester can activate the apoptotic factor Caspase-3 by activating the JNK/p53 signaling pathway, and Caspase-3 initiates the apoptotic process, triggering kidney injury [21]. Additionally, the enzymes related to the metabolisms of amino acid, lipid and carbohydrate in endogenous metabolism can be altered in response to 3-MCPD treatment. Meanwhile, proteins involved in these pathways can be also affected, mainly including oxidative phosphorylation, oxidative stress, apoptosis and autophagy [22].

As a branch of metabolomics, lipidomics emerged as a complementary approach to detect lipids and their synthesizing enzymes in biological systems, which can contribute to a better understanding of cellular lipid metabolism [23]. Based on a research model with high-throughput analytical techniques, lipidomics systematically analyzes changes in lipid composition and expression, the function and role of lipid molecules in various biological processes in organisms, and illustrates the relevant biological processes and mechanisms [24]. Studies demonstrated that 3-MCPD intake could induce oxidative stress in the kidney [11]. Reactive oxygen species (ROS) can attack unsaturated fatty acids in the body and produce lipid peroxides [25,26]. In addition, lipids are important components of cells and are involved in the composition of cell membranes and numerous organelle membranes. However, no lipidomics-based studies explored the mechanisms of 3-MCPD-induced nephrotoxicity, and few studies discussed the potential lipid biomarkers of 3-MCPD-induced kidney injury. Therefore, this study aimed to explore the effects of 3-MCPD on renal lipids and to explore potential risks and lipid biomarkers for various metabolic diseases caused by 3-MCPD exposure.

## 2. Materials and Methods

### 2.1. Chemicals and Reagents

3-Chloropropane-1,2-diol (>98%, 3-MCPD) was purchased from E. Merck (Darmstadt, Germany). LC-MS grade water was obtained from Milli-Q plus ultrapure water system (Billerica, MA, USA). Paraformaldehyde was purchased from Aladdin (Shanghai, China). H&E staining kits were purchased from Servicebio (Wuhan, China). Reagents used for lipidomic analysis, including methanol, chloroform, acetonitrile and isopropanol, were purchased from Thermo Fisher (Waltham, MA, USA).

### 2.2. Animal Experiments

Male Sprague Dawley (SD) rats (approximately 190–210 g) of 7–8 weeks old (animal certificate number SCXK (Xiang) 2019-0004) were purchased from Hunan Silaikejingda Laboratory Animal Technology Co., Ltd. (Changsha, China). Additionally, the study was compiled with the National Guidelines for Experimental Animal Welfare (GB/T 35892-2018) and the protocol was approved by the Animal Care and Use Committee of Nanchang University.

The rats were maintained in a controlled environment (temperature, 25 °C, constant humidity, 70%) with a 12 h light/dark cycle. The rats were provided with adequate food and water. After 7 days acclimatization, the rats were divided into the control group (CK) and the toxicity groups. The toxicity groups include the high dose group (MCh) and the low dose group (MCl). Gavage is an invasive method of drug administration that may elicit stress responses in test rats. Therefore, a control group was established for this study as a comparison. The rats in the toxicity group received 1 mL of 3-MCPD solution by gavage at a fixed time every day, while those in the control group received an equivalent volume of normal saline orally at the same time each day. The rats in the MCh and the MCl were given 45 mg/kg and 30 mg/kg 3-MCPD by gavage according to body weight, respectively. The dose of gavage was determined based on the previous studies [17,18]. The rats were kept in each group for 28 days and weighed every 4 days.

After 28 days, the rats were fasted for 12 h and were executed by isoflurane anesthesia after blood sampling. The kidneys were dissected from the rats, rinsed the external blood with pre-cooled saline. The saline was wiped on the kidneys, which were then weighed. The serum and tissues were used for biochemical analysis and other assays.

### 2.3. Measurement of Kidney Index

The kidneys of the rats were removed, rinsed with saline to remove blood and excess tissue was removed. The surface of the kidneys was then blotted out with clean paper towels. Finally, the kidneys were weighed and the organ index was calculated. The values were converted to organ index via the formula: organ index = kidneys weight (g) × 100/body weight of rat (g).

### 2.4. Histopathology Analysis

For the histopathology analysis, the kidney samples were cut into slices and exposed to 10% neutral formalin solution for at least 24 h. The histologic changes in kidney were examined after hematoxylin and eosin (H&E) staining of the sections. First, the slices were stained with a hematoxylin solution for 3–5 min, and the slices were then treated with a hematoxylin differentiation solution and hematoxylin Scott tap bluing. After being rinsed with water, the slices were sequentially dehydrated in 85% and 95% alcohol, respectively, for 5 min. Then, the slices were stained with eosin dye for 5 min. Finally, the slices were dehydrated in anhydrous ethanol 3 times and in xylene twice, before being sealed with neutral gum. The histological changes of the kidney were evaluated using a real-time digital pathology system (200×) (Aprio LV1, Leica Microsystems Ltd., Wetzlar, Germany).

### 2.5. Kidney Function

Serum creatinine and serum urea nitrogen were measured as the renal function markers using an automatic biochemical analyzer (Mindray BS-380, Shenzhen, China). The blood samples were centrifuged at 3000× *g* for 10 min at 4 °C. The serum obtained was used to determine renal function.

### 2.6. Evaluation of Oxidative Stress in the Kidney

The homogenate of kidney tissue (10% (*W*/*V*)) was prepared with 0.9% ice-cold phosphate buffer (pH 7.4). The homogenate was divided into two aliquots, one was centrifuged at 12,000 rpm for 10 min and the other at 4500 rpm for 10 min. The supernatant collected with the higher centrifugal speed was used to determine the malondialdehyde (MDA) and total antioxidant capacity (T-AOC) using commercial kits (purchased from Shanghai Beyotime Biotechnology, Shanghai, China). Additionally, the supernatant obtained with the lower centrifugal speed was used to determine the glutathione (GSH) using commercial kits (purchased from Nanjing Jiancheng Institute of Biological Engineering, Nanjing, China). The renal protein concentration was determined by using bicinchoninic acid (BCA) protein quantitative assay kit (purchased from Shanghai Beyotime Biotechnology, Shanghai, China).

### 2.7. Lipidomics Analysis

Lipids were extracted from kidney tissue according to chloroform–methanol method [27,28]. Briefly, the samples were homogenized with 750 μL chloroform–methanol mixture (2:1, *V*/*V*) (pre-cooled at −20 °C). After a 40 min ice bath, 190 μL water was added. The homogenate was placed on the ice for 10 min, after which the solution was centrifuged at 12,000 rpm for 15 min at room temperature. The lower layer of fluid was collected and then dried with a vacuum evaporator. The dried residue was reconstituted with 200 μL isopropanol, and 20 µL reconstituted solution was taken as the quality control (QC) sample. The sample was filtered with a 0.22 µm Millipore filter before being injected into UPLC-Q-Exactive/MS.

The separation was carried out on a Vanquish UPLC system (Thermo Scientific, Waltham, MA, USA) equipped with ACQUITY UPLC^®^ BEH C18 column (100 × 2.1 mm, 1.7 µm, Waters Corporation, Milford, CT, USA). The column temperature was kept at 50 °C. The autosampler temperature was 8 °C. A gradient program was applied at a flow rate of 0.25 mL/min and the injection volume was 2 μL. Mobile phase A consisted of acetonitrile/water (60:40, *V*/*V*) with 0.1%formic acid and 10 mM ammonium formate (A), and mobile phase B was isopropanol/acetonitrile (90:10, *V*/*V*) with 0.1% formic acid + 10 mM ammonium formate (B). An increasing linear gradient of solvent A (*V*/*V*) was used as follows: 0~5 min, 70~57% A; 5~5.1 min, 57~50% A; 5.1~14 min, 50~30% A; 14~14.1 min, 30% A; 14.1~21 min, 30~1% A; 21~24 min, 1% A; 24~24.1 min, 1~70% A; 24.1~28 min, 70% A.

The lipids detection was carried out on the Q-Exactive Focus mass spectrometer (Thermo Scientific, Waltham, MA, USA) with the spray voltage of 3.5 kV and 2.5 kV in positive and negative modes, respectively. Sheath gas and auxiliary gas were, respectively, set to 30 and 10 arbitrary units. The temperature of capillary was 325 °C. The Orbitrap analyzer scanned over a mass range of *m*/*z* 150–2000 for full scan at a mass resolution of 35,000. Data dependent acquisition MS/MS experiments were carried out using HCD scan. The normalized collision energy was set to 30 eV. Finally, dynamic exclusion was performed to remove unnecessary information in the MS/MS spectra.

### 2.8. Statistical Analysis

All data are expressed as mean ± standard deviation (SD). The statistical significance analysis was performed by SPSS 16.0 (SPSS Inc., Chicago, IL, USA). ANOVA for multiple comparisons was used to identify the significance of differences (*p* < 0.05) among groups. For lipidomic analysis, “Lipid searchTM” was used to identify lipid species based on LC-MS/MS. Lipid Search software (Version 4, Thermo Scientific, Pleasanton, CA, USA) was applied to process the raw data for lipid annotation to gain the results of mass to change ratio, retention time, intensity, etc. Subsequently, the peak pairs, the retention time correction, the extraction of the peak region and the analysis method were verified. The br.t. tolerance was set to 0.25, and the m-score threshold was set to 3.0. The processed data were normalized and integrated by the Perato scaling method, and principal component analysis (PCA) and partial least squares-discriminant analysis (PLS-DA) were performed by the software package R. The R software was also used for one-dimensional statistical analysis, including Student’s *t*-test, fold change analysis and hierarchical cluster analysis. Additionally, pathway analysis was performed by MetaboAnalyst 5.0 (www.metaboanalyst.ca) (accessed on 20 December 2022).

## 3. Results and Discussion

### 3.1. Body Weight and Kidney Weight

No rat deaths were observed during 28 days of repeated-dose treatment with 3-MCPD. Table 1 illustrates the changes in body weight of the rats during the 28-day culture period. The weight of rats in each group increased continuously over the 28-day period. The weight gain of rats in the CK group was significantly higher than that of the MCl group and the MCh group (*p* < 0.05), while there was no significant difference between the MCl group and the MCh group (*p* > 0.05). The organ index of the kidney changed significantly in the MCl group and MCh group, compared with the CK group (*p* < 0.01) (Table 1). It is worth mentioning that the kidney index was significantly higher in the MCh group than that in the MCl group (*p* < 0.01), which represented a dose-dependent effect of 3-MCPD on the kidney. These results indicated that the intake of 3-MCPD interfered with the body weight gain in rats, and also indicated that the kidney can be affected by the toxic effects of 3-MCPD, which was consistent with the results observed in previous experiments [20,29,30].

### 3.2. Changes in Serum Biochemistry Parameters

**Figure 1:**
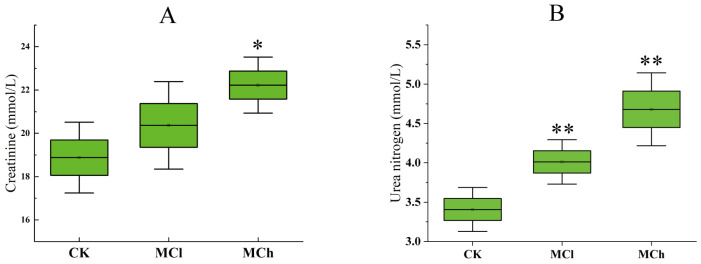
Effects of 3-MCPD on serum creatinine (**A**) and urea nitrogen (**B**) levels. Data are expressed as mean ± SD (*n* = 6). Significant difference (compared with the CK group) was indicated with ‘*’ or ‘**’. ‘*’ means *p* < 0.05, ‘**’ means *p* < 0.01.

Serum creatinine and urea nitrogen levels were altered in rats in both the MCh and MCl groups compared with the CK group (Figure 1). In the MCh group, the levels of serum creatinine (22.23 ± 1.18 mmol/L) and urea nitrogen (4.68 ± 0.42 mmol/L) were significantly increased compared with the CK group (*p* < 0.05). In the MCl group, the levels of serum creatinine (20.37 ± 1.84 mmol/L) in rats did not increase significantly compared with the CK group (*p* > 0.05), while the urea nitrogen (4.01 ± 0.26 mmol/L) levels exhibited a significant increase (*p* < 0.05). The results indicate that the effect of 3-MCPD on serum creatinine and urea nitrogen levels in rats is dose-dependent. Creatinine and urea nitrogen are important markers for evaluating whether the kidney function is normal [17]; elevated serum creatinine and urea nitrogen levels also indicate that 3-MCPD causes impairment of renal function in rats.

### 3.3. Levels of Oxidative Stress

The appearance of inflammation is associated with the excessive production of free radicals [31]. Free radical-induced lipid peroxidation leads to the accumulation of toxic by-products such as aldehydes and ketones, which, in turn, promote the production of free radicals and exacerbate oxidative stress [32]. Skamarauskas et al. and Steiner et al. found that the cellular damage was caused by oxidative stress induced with 3-MCPD in kidney cells [33,34]. Meanwhile, the proteomics and the transcriptomics analysis showed that 3-MCPD induced oxidative stress and altered GSH metabolism in the rat kidney [29,35,36,37].

**Figure 2:**
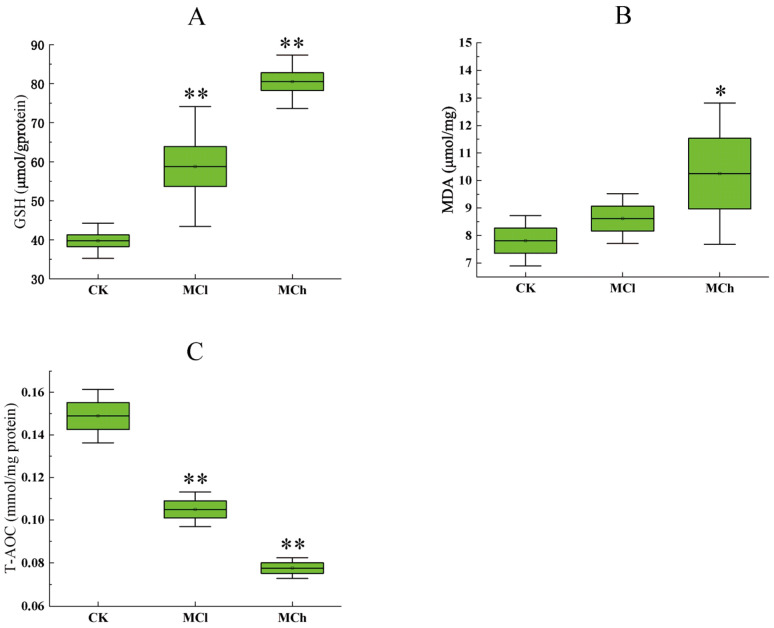
The level of GSH (**A**) and MDA (**B**) and the activity of kidney T-AOC (**C**) in the CK group and the 3-MCPD groups. Data are expressed as the mean ± SD of 6 rats per group. Significant difference (compared with the CK group) was indicated with ‘*’ or ‘**’. ‘*’ means *p* < 0.05, ‘**’ means *p* < 0.01.

In this study, levels of MDA, GSH and T-AOC in rat kidney were measured to assess the influences of oxidative stress induced by 3-MCPD. The renal GSH level in rats increased significantly in both the MCl and MCh groups, compared with the CK group (*p* < 0.05), and the level of GSH in the MCh group was obviously higher than that in the MCl group (*p* < 0.05) (Figure 2A). The increase in GSH levels may be due to the normal stress response for oxidation in the cells. In response to oxidative stress, cells can elevate GSH levels by means of increased synthesis. A previous metabolic study showed that GSH-type coupled compounds are typical metabolites of 3-MCPD esters, suggesting that GSH could be directly involved in the metabolism of 3-MCPD esters in vivo [38]. Therefore, the increase in GSH level in rat kidney tissue should be attributed to the self-protection mechanism of cells against oxidative stress injury. Regarding MDA, as shown in Figure 2B, the level in the MCh group exhibited a significant increase compared with the CK group (*p* < 0.05), while there was no significant difference in the levels between the CK group and the MCl group (*p* > 0.05). In Figure 2C, the levels of T-AOC in both the MCl group and the MCh group were observed to be lower than that in the CK group (*p* < 0.05). MDA was the end product of lipid peroxidation under the action of free radicals and can reflect the degree of lipid oxidation in the cell. The decrease in T-AOC and the increase in MDA indicated increased levels of oxidative stress in rats exposed to high dose of 3-MCPD. Moreover, the damage to renal antioxidant capacity was associated with the dose of 3-MCPD exposure.

### 3.4. Histopathological Analysis

**Figure 3:**
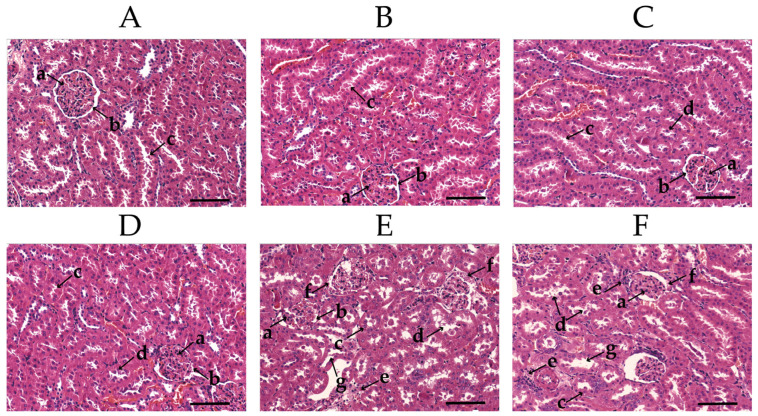
Kidney histopathology analysis of rats (H&E staining). Magnification: 200×, (**A**,**B**) CK, (**C**,**D**) MCl, (**E**,**F**) MCh. (a) glomerulus, (b) Bowman’s capsule, (c) kidney tubule, (d) renal tubular epithelial cell detachment, (e) interstitial inflammation, (f) eosinophilic material, (g) tubular dilatation. Scale bar in (**A**–**F**) 100 μm.

The H&E staining reflects the renal injury in rats after ingestion of 3-MCPD (Figure 3). In the CK group, the clear and intact structure of glomeruli and tubules were observed (Figure 3A,B). In the MCl group, slight detachment and minor misalignment of the renal tubular epithelial cells were observed, while the glomerular structure was observed intact (Figure 3C,D). In the MCh group, detached tubular epithelial cells and dilated tubules were observed, and inflammatory cell infiltration could be found in the tubular interstitium; furthermore, missing glomerular structures and eosinophilic material were also observed (Figure 3E,F). Compared with the MCl group, the kidneys of MCh rats encountered more severe damage. The renal tubular and glomerular structures were severely damaged and eosinophilia was observed in glomerulus. Moreover, inflammatory cell infiltration was present in the kidneys of rats in the MCh group. This was also mentioned in the previous study [39], which showed that inflammatory response is a typical characteristic feature of 3-MCPD-induced kidney injury.

### 3.5. Renal Lipidomics Analysis

**Figure 4:**
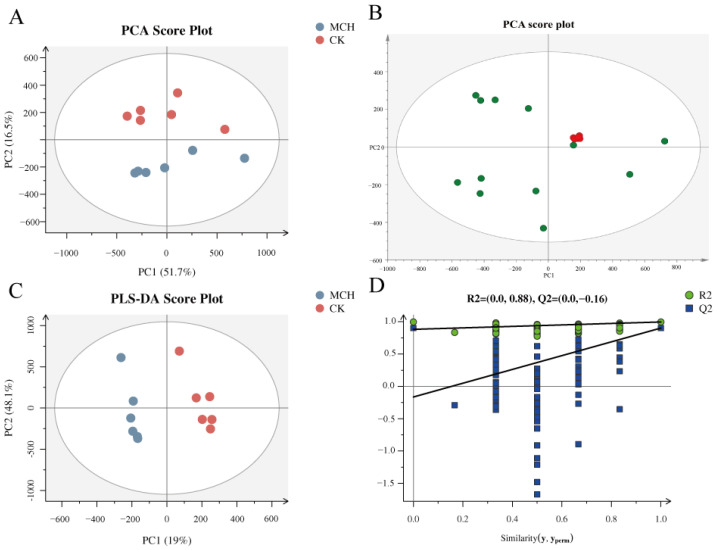
Kidney lipidomics multivariate statistical analysis. (**A**) PCA Score Plot of the MCl group and the MCh group, (**B**) PCA score of the total kidney lipid sample, and the red spots represent the QC samples, (**C**) PLS-DA Score Plot of the MCl group and the MCh group, (**D**) Placement test of kidney lipids in a PLS-DA model.

As a series of hydrophobic and amphiphilic molecules, lipids form the basic structure of cells and regulate a number of homeostatic processes. Disturbances in lipid metabolism may contribute to the accumulation of incompletely oxidized lipids, resulting in metabolic imbalances. In order to evaluate the effect of 3-MCPD on kidney lipids in rats and to identify the potential biomarkers, an untargeted lipidomics analysis was performed in both positive and negative modes on an UPLC-Q-Exactive-MS platform. To evaluate the stability of experimental data and the differences between the groups, the multivariate statistical analysis was carried out by principal component analysis (PCA). The QC samples and experimental samples were analyzed by PCA. As shown in Figure 4B, the QC samples were clustered together in the PCA diagram, indicating that the lipidomics analysis method based on LC-MS was stable, with reproducible and reliable data.

In the PCA score plot (Figure 4A), a clear separation was observed among the 3-MCPD group and the control group, suggesting that the lipids significantly changed in the kidney of rats treated with 3-MCPD. In addition, partial least-squares discriminant analysis (PLS-DA) was performed to understand the global lipid alterations and to evaluate the reliability of model. Good separations were observed in the mode of CK group versus 3-MCPD groups (Figure 4C). The stability and predictability of the model were good (R2X = 0.778, R2Y = 0.996 > 0.5, Q2 = 0.902 > 0.5) and the model was not over-fitted (R2 intercept was 0.91, Q2 intercept was −0.19 < 0) (Figure 4D). Taken together, these results indicate that the model is robust and reliable, with a good fit and significant differences between the 3-MCPD group and the control group for kidney lipids.

**Figure 5:**
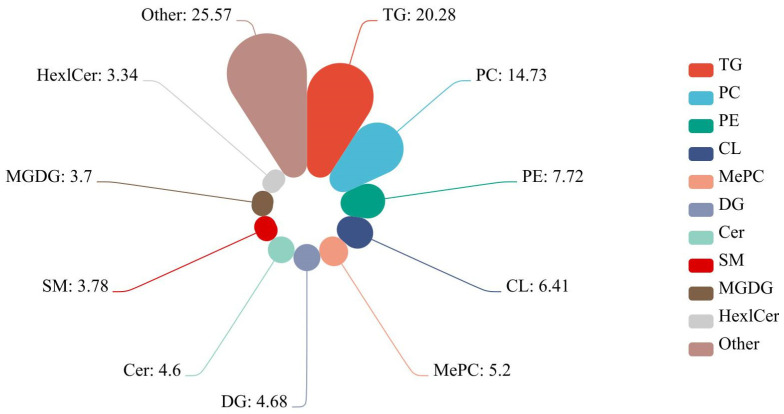
Lipid species distribution map (%).

A total of 3653 lipids were identified in the kidney tissues from the CK and 3-MCPD groups, including 741 triacylglycerols (TGs), 538 phosphatidylcholines (PCs), 282 phosphatidylethanolamines (PEs), 234 cardiolipins (CLs), 192 MePCs, 171 diacylglycerols (DGs), 168 ceramides (CERs), 138 sphingomyelins (SMs), 135 monogalatosyl diglycerides (MGDGs), 122 hexoside ceramides (HexCers) and others (Figure 5, Table S1). After statistical analysis, 183 significantly changed lipids were found (*p* < 0.05 and VIP (variable importance for the projection) ≥ 1) (Table S2). Of these, 83 lipids were upregulated and 100 were downregulated. The identified species can be grouped into 19 classes, including PC, PE, SM, TG, etc.

### 3.6. Potential Biomarker Analysis

**Figure 6:**
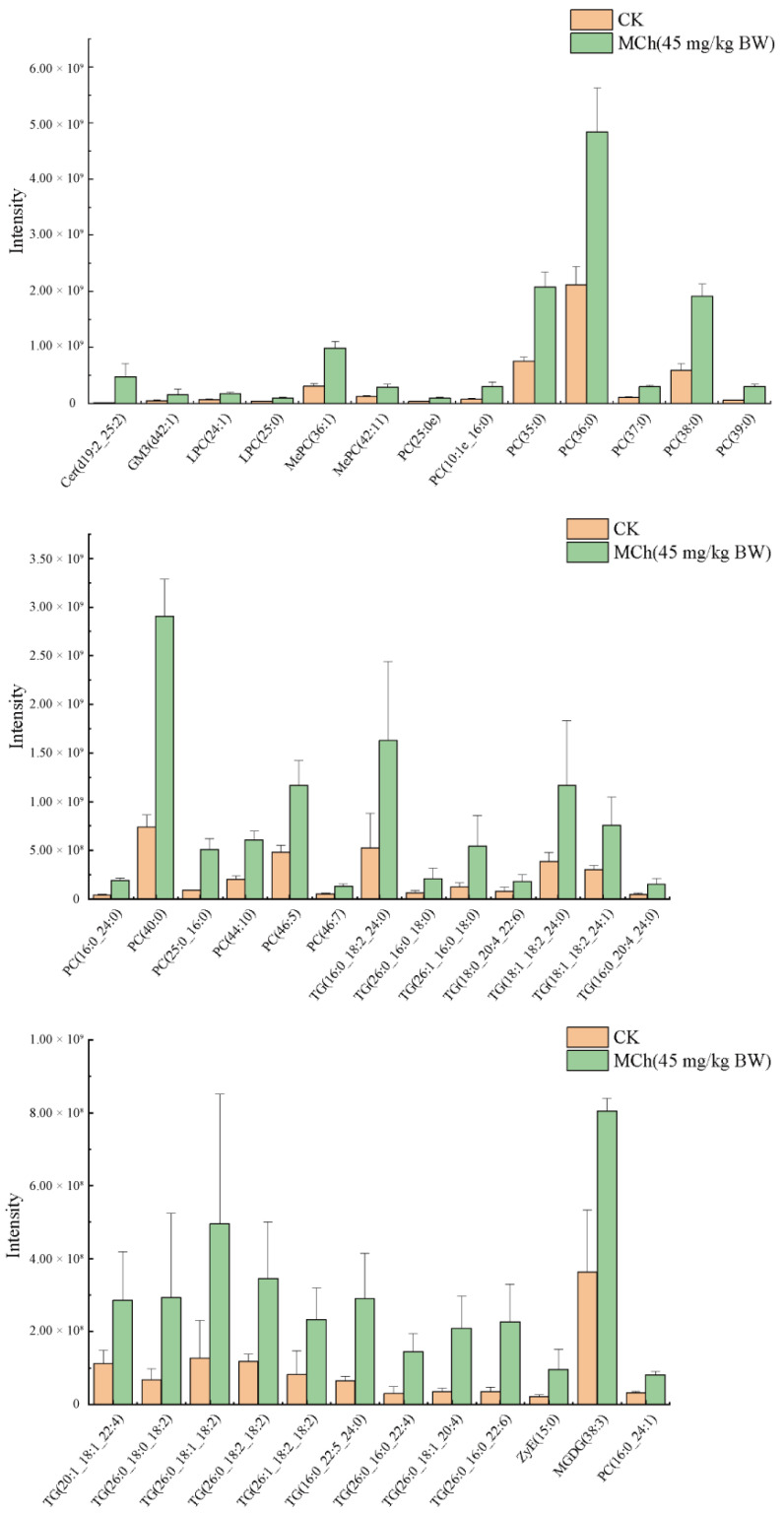
Lipid biomarker composition intensity in the kidney (potential lipid biomarkers were selected with the criterial of VIP ≥ 1, FC ≥ 2 or ≤0.5, *p* < 0.05 and AUC > 0.5.).

To find potential biomarkers of kidney injury induced by 3-MCPD, differentially regulated lipids were selected based on set criteria (VIP ≥ 1, Fold change ≥ 2 or ≤0.5 and *p* < 0.05). Based on the criteria, 49 lipids were found, including 38 upregulated lipids and 11 downregulated lipids. Meanwhile, receiver operating characteristic (ROC) curve was used to further find the potential biomarkers. The area under curve (AUC) greater than 0.5 was used as the criteria to find potential biomarkers. Eventually, 38 lipids were identified as the potential biomarkers (Table 2), including 16 TGs, 14 PCs, 2 LPCs, 2 MePCs, 1 GM3, 1 CER, 1 MGDG and 1 ZyE, which were upregulated (Figure 6).

**Figure 7:**
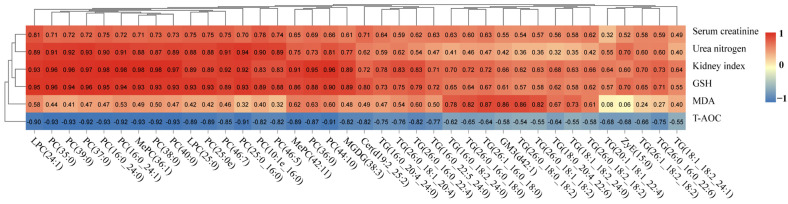
Pearson’s correlation between the potential lipid markers and the important metabolic parameters.

As mentioned earlier, oxidative stress and metabolic dysregulation in the kidney are both associated with lipid metabolism disorders. Oxidative stress is closely related to the 3-MCPD uptake process, and sustained oxidative stress can cause further kidney damage. The correlations between the lipid markers and the important metabolic parameters are shown in the Pearson’s correlation heatmap (Figure 7). Each lipid marker exhibited positive correlations with serum creatinine, urea nitrogen, MDA, GSH and kidney index. However, the T-AOC levels showed a negative correlation with each lipid marker. The expression of these lipids was positively correlated with the phenotype of kidney injury induced by 3-MCPD.

### 3.7. Pathway Analysis

**Figure 8:**
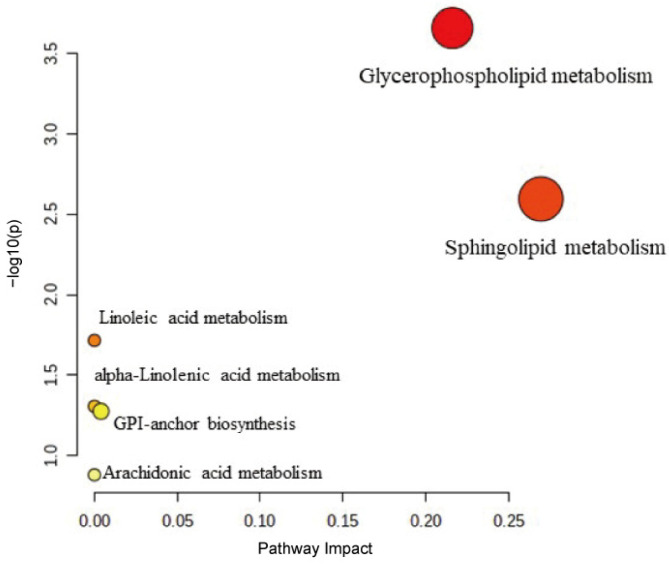
Lipid metabolic pathway analysis of different lipid species identified in the CK vs. the MCh.

In order to identify the pathway changes associated with lipid metabolism, pathway enrichment analysis was performed. Pathway enrichment analysis can provide some clues to the biochemical and signal transduction pathways that the significantly differentiated lipid species might be involved in [40]. Six pathways were found in our study (Figure 8, Table 3). The pathways of glycerophospholipid metabolism, sphingolipid metabolism, linoleic acid metabolism, alpha-Linolenic acid metabolism, glycosylphosphatidylinositol (GPI)-anchor biosynthesis and arachidonic acid metabolism were interfered in rats treated with 3-MCPD. The most critical lipid pathways were those with impact values > 0.1 [41]. Therefore, glycerophospholipid metabolism and sphingolipid metabolism were considered as the potential pathways.

Glycerophospholipids (GPs) are signaling molecules and energy sources for cells, and play an important role in the material transport, energy conversion and information transfer in biological membranes [42]. The lipidomics results of this study showed that the GP metabolites were significantly altered in the MCh group compared to the CK group. PE and PC are the two most abundant glycerophospholipids in eukaryotic cells and are the main structural and functional components of cell membranes [43]. We observed that PC (25:0_16:0), PC (39:0), PC (16:0_24:0) and PC (10:1e_16:0), etc., were significantly upregulated, while PC (15:0_20:3), PC (31:2e) and PC (18:3e_15:0), etc., were significantly downregulated in the kidney of rats treated with 3-MCPD. In addition, in the MCh group, PE (18:0_20:2), PE (20:0p_18:2) and PE (18:0_16:0) increased significantly, and a number of PEs, including PE (16:0_16:0), PE (16:0_20:5) and PE (34:0e), were significantly decreased. In addition, LPC (25:0) and LPC (24:1) were also significantly upregulated after 3-MCPD treatment. LPC can induce lipid peroxidation and damage endothelial cells [44]. Han et al. also confirmed that LPC is an important death effector of apoptosis [45].

Sphingolipids (SLs) have the important substructures to constitute lipid bilayers [46], and also act as the signaling molecules in mammalian cells [47]. SLs are associated with apoptosis and proliferation. The present research found that Cer (d19:2_25:2) and Cer (m17:1_25:3) were significantly upregulated and Cer (t18:0_20:0), Cer (t18:0_22:0) and Cer (d18:1_16:0) were significantly downregulated in the MCh group. In addition, SM(d35:2) was significantly upregulated and SM(d40:7), SM(t38:6), SM(t40:6), etc., were significantly downregulated. In addition to SM and Cer, some of HexCer were also altered. The alteration of HexCer levels indicate the abnormal regulation of ceramide synthase. Ceramide synthase is an important mediator of SL metabolism and a key regulator of endoplasmic reticulum (ER) homeostasis [48]. Additionally, one of the important pathways of apoptosis is the endoplasmic reticulum pathway [22]. In this study, Hex1Cer (t20:0_24:0+O), Hex1Cer (d18:1_21:3), Hex1Cer (d18:1_24:0) and Hex1Cer (t18:0_24:0+O) were found to be increased, Hex1Cer (t18:0_22:0+O) and Hex1Cer (t18:0_20:0+O) were observed to be decreased. Those alterations can result in homeostasis of the endoplasmic reticulum, and in turn activates the endoplasmic reticulum pathway of apoptosis.

Cell membranes act as the barrier between the cell and the external world, regulating the exchange of substances between the cell and the external world and maintaining a stable environment for normal cellular functions. Both glycerophospholipids and sphingolipids are the important composition of cell membrane. In the present research, 3-MCPD intake significantly altered the levels of glycerophospholipids and sphingolipids in rats. These changes lead to disturbances in glycerophospholipid metabolism and sphingolipid metabolism in the rat kidney, which disrupts the physiological structure of the cell membrane and leads to damage to cellular material exchange and signaling functions. In addition, 3-MCPD intake induced oxidative stress in the rat kidney. It was shown that ROS attacks unsaturated fatty acids in the body and produces lipid peroxides, resulting in a disruption of the physiological state of cell membranes in kidney tissue. Oxidative stress also induces lipid peroxidation in the cell membrane, decreasing the fluidity and increasing the adhesion of cell membrane to the endothelial cells, thus causing cell death. The production of oxidative stress causes a reduction in the antioxidant capacity of kidney tissue and the inactivation of key intra-cellular transporter enzymes [25,26]. Therefore, we can speculate that the intake of 3-MCPD could damage the cell membrane structure and affect cell membrane functions such as substance transport and signaling, which, in turn, cause damage to the cell. Similar conclusions were obtained in a study by Yang et al. [20], which indicated that the expression of ion transport-related proteins were significantly disrupted. The disruption of ion transport leads to the accumulation of ions, such as potassium and sodium ions in the cells, ultimately causing apoptosis. Later, other scholars also illustrated that 3-MCPD intake could induce the alterations in proteins involved in apoptosis and oxidative stress in the rat kidney [29].

Mitochondrial pathway is the major approach to cell apoptosis. Currently, numerous studies showed that 3-MCPD-induced kidney injury may be associated with mitochondria [17,18,49]. Phospholipids constitute the majority of the mitochondrial lipid structure, especially PC and PE. Approximately 95% of the lipids in the mitochondrial membrane are phospholipids. Alterations in phospholipid levels on mitochondria may impair the activity of key transmembrane proteins, leading to mitochondrial dysfunction and ultimately to apoptosis [50]. The large-scale alteration of phospholipids also confirms that the intake of 3-MCPD may affect mitochondrial function. Therefore, we can also speculate that the disruption of phospholipid metabolism caused by 3-MCPD intake may impair mitochondrial function, which, in turn, may lead to apoptosis.

In summary, the intake of 3-MCPD affected the normal glycerophospholipid metabolism and sphingolipid metabolism of the organism, leading to cellular oxidative stress. We speculate that the 3-MCPD intake disturbed the normal cell membrane function, causing disruption of ion transport and signal transduction in the cell membrane, leading to the accumulation of ions in the cell. In addition, the normal functions of organelles such as mitochondria and endoplasmic reticulum were also affected, resulting in the activation of the mitochondrial and endoplasmic reticulum routes of apoptosis.

## 4. Conclusions

In conclusion, our research revealed that 3-MCPD intake affects both body weight and renal index in rats and also confirmed that 3-MCPD intake induces an oxidative stress response in kidney cells. Lipidomics analysis revealed that the ingestion of 3-MCPD disrupts GP metabolism and SL metabolism, which are two main pathways of apoptosis induced by 3-MCPD. These discoveries also provide support for the findings of some previous studies. In addition, 38 lipid biomarkers were identified, including 16 TGs, 14 PC, 2 LPC, 2 MePC, 1 GM3, 1 CER, 1 MGDG and 1ZyE. This study can contribute to a better understanding of the renal toxicity of 3-MCPD.

## Figures and Tables

**Table 1:** Effects of 3-MCPD treatment on body weight and kidney weight in rats.

Items	CK	MCl	MCh
Initial BW (g)	249.62 ± 7.64	246.26 ± 2.82	247.80 ± 5.58
Final BW (g)	464.57 ± 41.70	406.29 ± 23.21	402.49 ± 29.46 *
BW gain (g)	214.95 ± 36.18	160.03 ± 22.29	154.70 ± 25.61 *
Kidney weight (g)	2.91 ± 0.24	3.70 ± 0.31 **	4.12 ± 0.47 **
Kidney index (%)	0.62 ± 0.01	0.89 ± 0.06 **	0.98 ± 0.04 **

BW: body weight. All data were expressed as mean ± SD (*n* = 6 rats/group). Significant difference (compared with the CK group) was indicated with ‘*’ or ‘**’. ‘*’ means *p* < 0.05, ‘**’ means *p* < 0.01.

**Table 2:** Identified Potential Biomarkers.

Lipids	AUC	Sig.	Lipid Name	AUC	Sig.
Cer (d19:2_25:2)	1	0.004	TG (16:0_18:2_24:0)	0.917	0.016
GM3 (d42:1)	1	0.004	TG (26:0_16:0_18:0)	0.889	0.025
LPC (24:1)	1	0.004	TG (26:1_16:0_18:0)	0.944	0.01
LPC (25:0)	1	0.004	TG (18:0_20:4_22:6)	0.861	0.037
MePC (36:1)	1	0.004	TG (18:1_18:2_24:0)	0.889	0.025
MePC (42:11)	1	0.004	TG (18:1_18:2_24:1)	0.889	0.025
PC (25:0e)	1	0.004	TG (16:0_20:4_24:0)	0.917	0.016
PC (10:1e_16:0)	1	0.004	TG (20:1_18:1_22:4)	0.861	0.037
PC (35:0)	1	0.004	TG (26:0_18:0_18:2)	0.917	0.016
PC (36:0)	1	0.004	TG (26:0_18:1_18:2)	0.889	0.025
PC (37:0)	1	0.004	TG (26:0_18:2_18:2)	0.917	0.016
PC (38:0)	1	0.004	TG (26:1_18:2_18:2)	0.917	0.016
PC (39:0)	1	0.004	TG (16:0_22:5_24:0)	0.944	0.01
PC (16:0_24:0)	1	0.004	TG (26:0_16:0_22:4)	0.972	0.006
PC (40:0)	1	0.004	TG (26:0_18:1_20:4)	1	0.004
PC (25:0_16:0)	1	0.004	TG (26:0_16:0_22:6)	0.972	0.006
PC (44:10)	1	0.004	ZyE (15:0)	0.889	0.025
PC (46:5)	0.944	0.01	MGDG (38:3)	1	0.004
PC (46:7)	1	0.004	PC (16:0_24:1)	1	0.004

**Table 3:** The Pathway Analysis of Different Lipid Species.

Pathway name	Total Metabolism	Hits	Raw p	−Log(P)	FDR	Impact
Glycerophospholipid metabolism	36	3	0.00022	3.66	0.02	0.21631
Sphingolipid metabolism	21	2	0.00254	2.60	0.11	0.26978
Linoleic acid metabolism	5	1	0.01923	1.72	0.54	0
alpha-Linolenic acid metabolism	13	1	0.04936	1.31	0.89	0
GPI-anchor biosynthesis	14	1	0.05307	1.28	0.89	0.00399
Arachidonic acid metabolism	36	1	0.13171	0.88	1	0

‘Total’ means all of the differential lipid species in the pathway, ‘Hits’ means different lipid species involved in the pathway. ‘Raw p’ means the raw P calculated from the enrichment analysis; ‘Impact’ is the pathway impact value calculated from pathway analysis. GPI: glycolphosphatidylinositol.

## Data Availability

The datasets generated during and/or analyzed during the current study are available from the corresponding author on reasonable request.

## Supplementary Materials

The following supporting information can be downloaded at: https://www.mdpi.com/article/10.3390/toxics11060479/s1, Table S1: Identified lipids; Table S2: Differential lipids.
